# Causal Influences of Same-Sex Attraction on Psychological Distress and Risky Sexual Behaviors: Evidence for Bidirectional Effects

**DOI:** 10.1007/s10508-022-02455-9

**Published:** 2022-11-04

**Authors:** Olakunle Ayokunmi Oginni, Kai Xiang Lim, Kirstin Lee Purves, Yi Lu, Ada Johansson, Patrick Jern, Frühling Vesta Rijsdijk

**Affiliations:** 1 The Social, Genetic and Developmental Psychiatry Centre, Institute of Psychiatry, Psychology and Neuroscience, King’s College London, Denmark Hill, London, SE5 8AF UK; 2 Department of Mental Health, Obafemi Awolowo University, Ile-Ife, Nigeria; 3 Department of Medical Epidemiology and Biostatistics, Psychiatry Genomic Institute, Karolinska Institutet, Stockholm, Sweden; 4 Department of Psychology, Åbo Akademi University, Åbo, Finland

**Keywords:** Same-sex attraction, Psychological distress, Risky sexual behavior, Mendelian Randomization, Direction of Causation, Sexual orientation

## Abstract

Although health disparities among same-sex attracted compared to heterosexual individuals are typically explained by minority stress, there is limited evidence for a causal effect. This study investigated whether same-sex attraction was causally associated with psychological distress and risky sexual behavior using sociosexual behavior as a proxy. The sample comprised monozygotic and dizygotic twins and their non-twin siblings (*n* = 2036, 3780 and 2356, respectively) genotyped and assessed for same-sex attraction, psychological distress (anxiety and depressive symptoms), and risky sexual behavior. Causal influences were investigated with same-sex attraction as the predictor and psychological distress and risky sexual behavior as the outcomes in two separate Mendelian Randomization-Direction of Causation (MRDoC) models using OpenMx in R. The MRDoC model improves on the Mendelian Randomization and Direction of Causation twin models by allowing analyses of variables with similar genetic architectures, incorporating polygenic scores as instrumental variables and specifying pleiotropy and residual covariance. There were significant causal influences flowing from same-sex attraction to psychological distress and risky sexual behavior (standardized coefficients = 0.13 and 0.16; 95% CIs 0.03–0.23 and 0.08–0.25, respectively). Further analyses also demonstrated causal influences flowing from psychological distress and risky sexual behavior toward same-sex attraction. Causal influences from same-sex attraction to psychological distress and risky sexual behavior may reflect minority stress, which reinforces ongoing measures to minimize social disparities. Causal influences flowing in the opposite direction may reflect rejection sensitivity, stigma-inducing outcomes of risky sexual behavior, and recall bias; however, further research is required to specifically investigate these processes.

**Supplementary Information:**

The online version contains supplementary material available at 10.1007/s10508-022-02455-9.

## Introduction

Sexual minority status (identifying as lesbian, gay or bisexual) which is characterized by varying degrees of same-sex sexual attraction and behavior (Geary et al., 2018) has been consistently associated with health disparities relative to heterosexual individuals. These include higher levels of psychological distress characterized by greater depression and anxiety symptoms (King et al., 2008; Plöderl & Tremblay, 2015; Semlyen et al., 2016). Furthermore, poorer sexual health indices such as sexually transmitted infections (Bränström & Pachankis, 2018; Charlton et al., 2011) are indicative of greater risky sexual behaviors such as higher lifetime and concurrent sexual partners, inconsistent use of condoms and sex under the influence of substances (Cabecinha et al., 2017; Poteat et al., 2019).

### Mechanisms of Health Disparities in Sexual Minorities

#### Minority Stress

**Fig. 1:**
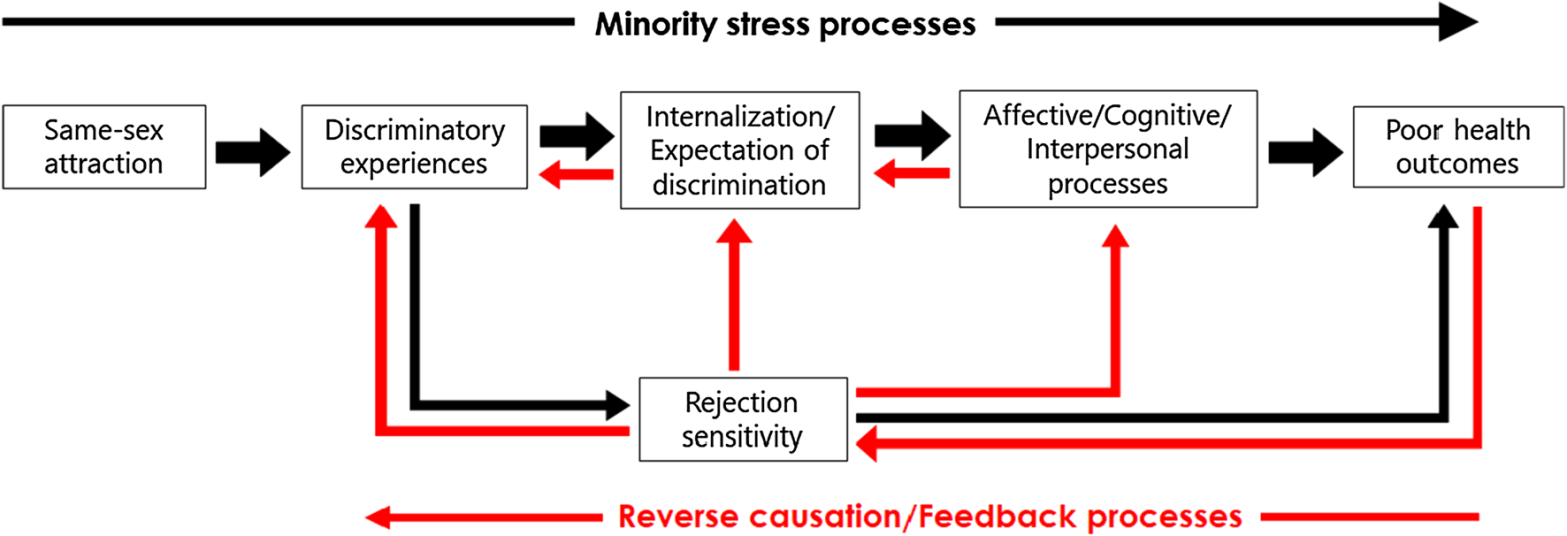
Schematic diagram illustrating possible causal pathways from same-sex attraction to poor health outcomes. Causal pathways from same-sex attraction toward poor health outcomes may involve minority stress processes (discriminatory experiences and the internalization and expectation of discrimination), affective, cognitive and interpersonal processes and rejection sensitivity (black arrows). Poor health outcomes may facilitate sexuality-related discrimination via reverse causal mechanisms such as through rejection sensitivity (red arrows). We describe causal influences as “flowing” from an exposure toward an outcome (e.g., see Minică et al., 2020) in recognition of intermediary processes which may simultaneously have downstream or upstream effects in the causal pathways

The minority stress framework suggests that disparities in mental wellbeing and risky sexual behaviors can be explained by stressors related to sexual minority status. These include discrimination, the expectation of prejudice, concealment of sexual orientation and internalized stigma (Fig. 1; Meyer, 2013; Newcomb & Mustanski, 2011; Rogers et al., 2018). Generally, stressors increase the likelihood of mental health problems by disrupting biological stress regulatory mechanisms (Lupien et al., 2009). In addition, the psychological mediation theory suggests that minority stressors may specifically disrupt emotional regulation and coping while promoting negative cognitive styles and social isolation (Hatzenbuehler, 2009). Another mechanism of minority stress is rejection sensitivity from repeated discrimination which increases the expectation of discrimination, negative interpretation of ambiguous or neutral events and negative emotional responses to actual and perceived discrimination (Feinstein, 2020). However, the evidence for these mechanisms in same-sex attracted persons is largely cross-sectional (Bailey, 2020; Feinstein, 2020; Hatzenbuehler, 2009) which precludes causal inference. Most of the longitudinal studies that have been conducted are observational and do not specify models appropriate for testing causal mechanisms (e.g., Bränström, 2017; Sarno et al., 2020). These studies are further limited by utilizing samples comprising only same-sex attracted individuals (e.g., Rosario et al., 2002) which may limit the capacity to identify mechanisms of disparities in health outcomes relative to heterosexual samples (Schwartz & Meyer, 2010).

#### Correlated Genetic and Environmental Influences

An alternative explanation for the observed associations between same-sex attraction and psychological distress and risky sexual behavior is the effect of shared etiological genetic and environmental influences. This is supported by evidence indicating that same-sex sexual behavior and non-heterosexual sexual orientation are genetically correlated with anxiety and depressive symptoms (Ganna et al., 2019; Zietsch et al., 2012) and more lifetime sexual partners (Burri et al., 2015; Ganna et al., 2019). These genetic correlations have previously been interpreted as indicating horizontal pleiotropy, i.e., genetic variants simultaneously influencing multiple genetic pathways; see, e.g., Zietsch (2011). However, findings from a recent study indicated vertical pleiotropy whereby genetic influences on sexual orientation are transmitted through separate phenotypic causal paths to mental health problems and risky sexual behavior (Oginni et al., 2020, 2022), but this study did not investigate the direction of causal effects.

#### Reverse Causation

A final possibility is reverse causation, whereby factors associated with adverse health indices increase the likelihood of experiencing stressful events associated with same-sex attraction (Bailey, 2020). For example, rejection sensitivity has been shown to generate dependent stressful life events (i.e., events resulting from an individual’s behavior such as interpersonal conflicts) which are in turn associated with adverse mental health outcomes (Liu et al., 2014). Moreover, strategies to protect against anticipated rejection or discrimination may paradoxically amplify such feelings (Fig. 1, London et al., 2012). Considering that rejection sensitivity may also be triggered by experiences related to discrimination based on same-sex attraction (Feinstein, 2020), these findings suggest the possibility that downstream processes may influence upstream ones in the pathogenesis of health disparities in same-sex attracted persons; however, this possibility has not been previously investigated.

A clearer understanding of the causal relationships between same-sex attraction and disparities in mental and sexual health can provide justification for existing social and legal interventions to minimize these disparities. For example, causal influences flowing from same-sex attraction toward adverse health outcomes may support frameworks such as the minority stress model and its extensions, and support policies aimed at reducing inequalities and discrimination based on sexual orientation. Similarly, significant reverse causal pathways would suggest the need for more research to explore explanatory mechanisms which can be incorporated into existing frameworks and inform future intervention design.

We, therefore, propose to use the novel Mendelian Randomization-Direction of Causation (MRDoC) model to determine whether causal influences flow from same-sex attraction toward increased psychological distress and risky sexual behavior using cross-sectional data. This model combines Mendelian Randomization (MR) which utilizes genetic variants as an instrument, with the Direction of Causation (DoC) twin model (Minică et al., 2018), both of which test unidirectional and bidirectional causation, respectively. Both models (which we discuss in more detail in the "Method" section) are each sufficient to determine the Direction of Causation using cross-sectional data under certain conditions (Burgess & Thompson, 2011; Heath et al., 1993; Tick et al., 2016), and the MRDoC allows these conditions to be relaxed while generating less biased estimates of causal influences compared to the more commonly used MR methods. Using data simulations, Minică et al. (2018) showed that both standard MR and MRDoC yielded similar estimates when there was no pleiotropy. However, in the presence of pleiotropy, standard MR overestimated causal path estimates even when there was no true effect. We discuss the MRDoC model and its limitations further in the Method and Limitation sections. Based on existing theory (Feinstein, 2020; Hatzenbuehler, 2009; Meyer, 2013), we hypothesized causal influences flowing from same-sex attraction to psychological distress and risky sexual behaviors. To explore reverse causation, a secondary objective of the present study was to investigate the possibility of causal influences flowing from psychological distress and risky sexual behavior toward same-sex attraction. Based on evidence that adverse psychological outcomes such as rejection sensitivity may generate dependent stressful life events (Liu et al., 2014) and exacerbate existing stress (London et al., 2012), we hypothesized reverse causal processes from psychological distress and risky sexual behavior flowing toward same-sex attraction.

## Method

### Sample

This comprised twin participants from the second wave of the Finnish Genetics of Sexuality and Aggression cohort and their siblings (Johansson et al., 2013). Families with twins were identified from the government-based registry of all Finnish citizens, and twins aged 18–33 years and siblings aged at least 18 years and resident in Finland at the time of data collection were invited to participate in the study. Of the 23,577 individuals invited by mail to participate in the study, 10,524 (6,531 twin individuals and 3993 siblings) responded giving a response rate of 45% which is comparable to rates from mail surveys (Guo et al., 2016). Only participants who responded to at least 80% of the items per variable were included in the present study and 143 twins with indeterminate zygosities were further excluded. This gave a total sample size of 8172 individuals (2036 and 3780 monozygotic and dizygotic twins, respectively, and 2356 siblings). Zygosity was determined using two questions about physical similarity (Sarna et al., 1978) with an accuracy of 91% as determined by genotyping a subset of the sample (Johansson et al., 2013). The DNA-determined zygosity was used if there was a discrepancy with the question-based zygosity in individuals who were genotyped.

Ethical approval was obtained from the Ethics Committee of the Department of Psychology, Åbo Akademi University, Finland, and informed consent was obtained from all participants.

### Measures

#### Covariates

These included age which was assessed using a single question and sex which was ascertained from the Central Population Registry.

#### Same-Sex Attraction

This was ascertained by two questions: 1. “How often have you on average felt interest toward a member of the same sex?” and 2. “If an attractive man (woman for female participants), whom you like, proposes sexual interaction to you, how probable is it that you could do it (if you decide activity and nobody would ever know)?” These were, respectively, scored on a 7-point Likert scale ranging from “Never” (0) to “Every day” (6) and a 6-point Likert scale ranging from “Impossible” (1) to “Very likely” (6). The Cronbach’s alpha for both questions in this study was 0.66.

#### Depressive and Anxiety Symptoms

These were assessed using the depression and anxiety subscales of the 18-item self-report Brief Symptom Inventory (Derogatis, 2001). Each subscale consists of six questions individually scored on a 5-point Likert scale ranging from “Not at all” (0) to “Extremely” (4) with possible scores for each subscale ranging between 0 and 24. Cronbach’s alphas for both subscales in the present study were 0.84 and 0.85, respectively, and scores in each subscale were summed and used in subsequent analyses with higher scores indicating higher levels of symptoms.

#### Risky Sexual Behavior

This was assessed using the Behavior subscale of the 7-item Sociosexual Orientation Inventory (SOI, Simpson & Gangestad, 1991) as a proxy. This subscale comprises three questions about the number of sexual partners in the past year, planned number of sex partners in the next five years and the number of onetime sexual partners. Responses were scored on a 9-point Likert scale ranging from “0” (0) to “20 or more” (8) and the sum of the responses used in subsequent analyses. Cronbach’s alpha for this measure in the present study was 0.68; its significant positive correlation with number of sexual partners at a time and lack of significant correlation with sexual drive indicate good convergent and discriminant validity, respectively (Simpson & Gangestad, 1991). Consistent with findings from an international review (Slaymaker, 2004), these indices support our use of this measure as a proxy for risky sexual behavior; higher scores were taken to indicate higher sexual risk.

#### Polygenic Risk Scores

Saliva samples for DNA extraction were obtained using the Oragene DNA collection kit (James et al., 2011). A total of 6428 kits were sent to participants who had indicated willingness to provide saliva samples, of which 4278 were returned (return rate was 66%; Johansson et al., 2013). Of these, 3768 samples were available for processing (up to 510 samples were no longer viable at the time of DNA extraction in 2020), of which 229 not be linked with phenotypic data. The remaining 3539 samples were genotyped using the Illumina GSA beadchip v3. Details of quality control for sample and SNPs (single nucleotide polymorphisms) are described in the supplement; altogether, 118 (3.3%) participants and about 10% of the SNPs were excluded.

The polygenic score for same-sex behavior was calculated by summing the number of alleles of each SNP associated with same-sex behavior across the genome based on results from a discovery genome-wide association study (GWAS) which included participants from the UK Biobank and 23andMe (Ganna et al., 2019). Each allele was weighted by the effect size of its association with same-sex behavior. Using a clumping and thresholding approach, we calculated polygenic scores by including SNPs at different genome-wide *p*-value thresholds (0 ≤ *p* ≤ 1, at intervals of 5 × 10^–5^) based on their *p*-values in the original GWAS. The score predicting the greatest target trait variance was selected for further analyses. For our main analyses, the target traits comprised the two same-sex attraction questions which were separately regressed on all possible polygenic scores while including age, sex and the first 10 principal components as covariates (Kerminen et al., 2019). The scores which predicted the greatest variance for each trait were used in subsequent analyses (Choi et al., 2020). While we note that this results in a polygenic score for same-sex behavior at two different thresholds (6.0 × 10^–4^ and 9.5 × 10^–4^, Supplementary Table S0) rather than two distinct polygenic scores, we have, respectively, designated these as polygenic scores for interest in same-sex sexual activity (PS_SSI_) and probability of same-sex sexual activity (PS_SSP_) for ease of reference. Data overfitting and multiple testing were adjusted for by carrying out 10,000 permutations at the best threshold for each trait. PRSice-2 was used for polygenic analyses and standard procedures (imputation quality > 0.9, and minor allele frequencies > 0:05) were applied (Choi & O'Reilly, 2019; Choi et al., 2020). The full GWAS summary statistics for the 23andMe discovery data set will be made available through 23andMe to qualified researchers under an agreement with 23andMe that protects the privacy of the 23andMe participants. Please visit https://research.23andme.com/collaborate/#dataset-access/ for more information and to apply to access the data.

#### Latent Factors

Four latent factors were specified to reduce measurement error, improve validity of the instruments and facilitate twin model-fitting analyses. These included one predictor: same-sex attraction (SSA, with the two questions assessing interest in and probability of same-sex sexual behavior as indicators); two outcomes: psychological distress (PD, with depressive and anxiety symptom scores as indicators) and risky sexual behavior (RSB, risky sexual behavior scores as the single indicator); and an instrument factor: Genetic propensity for same-sex attraction (PS_SSA_—with PS_SSI_ and PS_SSP_ polygenic scores as indicators). Although the 136 SNPs used in deriving PS_SSI_ are included among the 214 used in deriving PS_SSP_, the specification of both scores as indicators of a latent genetic propensity allowed the exclusion of any measurement or other error influences on the instrument factor.

### Statistical Analyses

Data cleaning and preparation were carried out using SPSS version 25 (IBM Corp, 2017) and OpenMx in R (Neale et al., 2016). Consistent with standard practice in twin studies (McGue & Bouchard, 1984), the mean effects of age and sex were regressed out of the study variables and the residuals normalized and used in subsequent analyses using OpenMx.

#### Phenotypic Models

##### Correlations of Variables and Latent Factors

Phenotypic correlations of the observed variables and latent factors were derived using maximum likelihood estimation in constrained correlational models with within-person correlations constrained to be equal across zygosity, birth order and sibship while cross-trait and cross-twin/cross-sibling correlations were constrained to be symmetrical. Separate factor correlation models were specified for the correlations of psychological distress and risky sexual behavior with same-sex attraction and its instrument.

#### Genetic Models

##### Multivariate Biometric Genetic Models

These were specified to resolve variable and factor variances into genetic (A) and shared and individual-specific environmental (C and E, respectively) influences using the Cholesky decomposition. This capacity of the classical twin design rests on the assumption that A, C and E influences are independent and are not affected by assortative mating; that monozygotic and dizygotic twin pairs raised together are, respectively, 100% and 50% genetically identical and are similarly influenced by their shared environments, but do not share E influences (Rijsdijk & Sham, 2002). Similar to dizygotic twins, sibling pairs are assumed to share their common environment to the same extent and are only 50% genetically similar; we therefore constrained dizygotic cross-twin correlations and cross-sibling correlations to be equal. The polygenic score variables and factor were excluded from these analyses because these variables are completely genetic and there was no logic for parsing their variances into A, C and E components. Separate biometric genetic models were specified for the component influences on the relationships involving depressive and anxiety symptoms, and those involving risky sexual behavior.

##### Mendelian Randomization—Direction of Causation (MRDoC) Models

These were specified to determine whether sexual orientation was causally associated with psychological distress and risky sexual behavior. The MRDoC model was described as a combination of the Direction of Causation (DoC) twin model and Mendelian Randomization (MR; Minică et al., 2018). While both rely on cross-sectional data, the DoC specifies hypothesis-free bidirectional causal paths between two variables and depends on both variables having different etiological architectures (e.g., ACE versus ADE; Heath et al., 1993; Tick et al., 2016) which is not always possible. In contrast, MR tests and estimates a hypothesis-driven unidirectional causal relationship by incorporating single genetic variants associated with the exposure as instrumental variables (Burgess & Thompson, 2011). An instrumental variable allows the inference of causation by controlling for endogeneity [i.e., residual exposure–outcome covariance, see Burgess & Thompson (2011)] and to be valid, it must be: i. associated with the exposure variable, ii. independent of confounders and iii. independent of the outcome after controlling for the exposure and other confounders (Burgess & Thompson, 2011; Minică et al., 2018). Analogous to experimental designs, the random allocation of genetic material during meiosis further provides randomization, which should confer independence of the genetic variant from the outcome effect (Davey-Smith & Ebrahim, 2003). However, single genetic variants in MR typically have small associations with exposure variables and are subject to weak instrument bias (Burgess & Thompson, 2011).

**Fig. 2:**
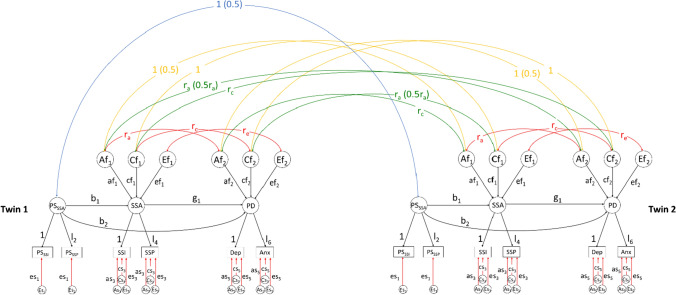
Path diagram illustrating the full Mendelian Randomization-Direction of Causation model for the relationship between same-sex attraction (SSA) and psychological distress (PD). PS_SSA_: Genetic propensity for same-sex attraction with polygenic scores for interest in same-sex sexual activity (PS_SSI_) and probability of future same-sex activity (PS_SSP_) as indicators. Path b_1_ is the instrumental path from PS_SSA_ to SSA and identifies the causal path g_1_ from SSA to PD, path b_2_ represents pleiotropic effects. *Af*_1_, *Af*_2_, *Cf*_1_, *Cf*_2_, *Ef*_1_ and *Ef*_2_ denote additive genetic and shared and individual-specific environmental influences on the variances of SSA and PD, respectively; *af*_1_, *af*_2_, *cf*_1_, *cf*_2_, *ef*_1_ and *ef*_2_ are their respective path coefficients. *r*_*a*_, *r*_*c*_ and *r*_*e*_ denote the correlation coefficients between the additive genetic and shared and individual-specific environmental factors on SSA and PD. *As*_3_, *As*_5_, *Cs*_3_, *Cs*_5_, *Es*_3_, *Es*_5_ denote variable-specific additive genetic and shared and individual-specific environmental influences on the variances of SSI (Interest in same-sex sexual activity); SSP (Probability of future same-sex sexual activity), Dep and Anx (Depressive and Anxiety symptoms, respectively). *Es*_1_ and *Es*_2_ denote the residual variances of PS_SSI_ and PS_SSP_, respectively; as polygenic scores are completely genetic, their variances were not decomposed into ACE variance components. The following constraints were specified to identify the measurement model: One unstandardized factor loading per latent factor was constrained to 1 to scale each latent factor while the other loadings (*l*_2_, *l*_4_ and *l*_6_) were freely estimated; the unstandardized variable-specific genetic and environmental influences were constrained to be equal across the two indicator variables per latent factor. This model was also specified for risky sexual behavior (RSB) as the outcome factor, but with one indicator; the residual variance constrained to 0 to identify the measurement model. The fixed correlation coefficients across dizygotic twins are in parentheses. The full unidentified model as depicted here was identified by dropping r_e_ between exposure and outcome (adapted from Minică et al., 2018)

The MRDoC model combines the DoC and MR models by specifying a unidirectional causal path in the DoC twin model. Furthermore, it overcomes weak instrument bias by incorporating polygenic scores as an instrument and adjusts for the violation of the third property of an instrument by specifying a pleiotropic path (*b*_2_, Fig. 2) alongside the instrumental and causal paths (*b*_1_ and* g*_1_, respectively; Minică et al., 2018). The under-identification which the specification of the pleiotropic path introduces in the full MRDoC model can be eliminated by dropping the E correlation paths r_e_ or the pleiotropic path (Fig. 2; Kohler et al., 2011; Minică et al., 2018). We retained the pleiotropic path despite the small magnitude of the path coefficient based on significant associations between the instrument and outcomes from preliminary analyses (Table S1, Table 2) and previous recommendation (Minică et al., 2020); and dropped the r_e_ path as is commonly done to identify causal paths in behavioral genetic models (Kohler et al., 2011; Minică et al., 2020). We further verified local identification using the *mxCheckIdentification* command in OpenMx. Thus, the MRDoC model allows investigation of the Direction of Causation using an instrument while adjusting for pleiotropy and residual covariance (Minică et al., 2020). A further advantage of the MRDoC model is its use with variables having similar etiological architectures in contrast to the traditional DoC model.

For the present study, two separate MRDoC models were specified to test and estimate the causal influences of same-sex attraction on psychological distress and risky sexual behavior (MRDoC models 1 and 2, respectively) with significant positive causal path coefficients indicating causal influences flowing from same-sex attraction to the outcomes.

##### Reverse Causation

We further specified two separate MRDoC models to investigate the possibility of reverse causation with PD and RSB as the respective predictors and same-sex attraction as the outcome (i.e., causal influences flowing from PD and RSB toward same-sex attraction). Genetic risk for psychological distress and risky sexual behavior (PS_PD_ and PS_RSB_, respectively) were specified as instruments: the indicators for PS_PD_ were polygenic scores for depressive and anxiety symptoms [constructed as previously described using summary statistics from GWASs by Howard et al. (2019) and Purves et al. (2020) with depressive and anxiety symptoms as the target traits, respectively], while those for PS_RSB_ were polygenic scores for number of sexual partners and risk behaviors [constructed using summary statistics from the GWAS by Linnér et al. (2018) with risky sexual behavior as the target trait for both scores].

### Secondary Analyses

#### Phenotypic Mendelian Randomization models

To compare findings from the MRDoC models with standard Mendelian Randomization (MR), we specified two separate phenotypic MR models with the genetic propensity for same-sex attraction (PS_SSA_) and same-sex attraction (SSA) factors as the instrument and exposure, respectively, and the psychological distress (PD) and risky sexual behavior (RSB) factors as outcomes in the first and second models, respectively. We specified pleiotropic paths as in the MRDoC models, but did not specify residual covariance as this would make the model unidentified.

## Results

### Descriptive Statistics

The mean ages of monozygotic and dizygotic twins were 24.9 (± 3.98) and 25.1 (± 4.01) years, respectively, while that for their non-twin siblings was 28.1 (± 4.78) years (Table 1). Eighty percent (6538) of the participants indicated never having had interest in same-sex sexual activity while 45% (3675) reported that future same-sex sexual activity was impossible; and these proportions were comparable in monozygotic and dizygotic twins and their siblings. The mean depressive and anxiety symptom scores were 4.8 (± 4.37) and 3.5 (± 3.91) while the mean risky sexual behavior score was 5.7 (± 4.57) in the whole sample.

**Table 1:** Descriptive statistics

Variables	MZ twins (*n* = 2036)		DZ twins (*n* = 3780)		Siblings (*n* = 2356)		Total (*n* = 8172)	
Continuous variables	Mean	SD	Mean	SD	Mean	SD	Mean	SD
Age	24.9	3.98	25.1	4.01	28.1	5.73	25.9	4.78
Depressive symptoms	4.6	4.33	4.99	4.39	4.72	4.36	4.8	4.37
Anxiety symptoms	3.4	3.91	3.7	3.97	3.3	3.79	3.5	3.91
Risky sexual behavior	5.4	4.54	5.9	4.69	5.7	4.39	5.7	4.57
SSI^a^	0.32	0.93	0.38	1.01	0.33	0.84	0.35	0.94
SSP^a^	2.19	1.55	2.33	1.59	2.32	1.57	2.29	1.58

Categorical variables	*n*	%	*n*	%	*n*	%	*n*	%
Sex								
Male	658	32.3	1376	36.4	824	35.0	2858	35.0
Female	1378	67.7	2404	63.6	1532	65.0	5314	65.0
SSI^b^								
0	1665	81.8	3009	80.1	1864	79.1	6538	80.0
1	259	12.7	507	13.4	365	15.5	1131	13.8
2	47	2.3	121	3.2	70	3.0	238	2.9
3	14	0.7	41	1.1	15	0.6	70	0.9
4	17	0.8	24	0.6	14	0.6	55	0.7
5	8	0.4	16	0.4	8	0.6	32	0.4
6	26	1.3	62	1.6	20	0.8	108	1.3
SSP^b^								
1	995	48.9	1670	44.2	1010	42.9	3675	45.0
2	466	22.9	900	23.8	605	25.7	1971	24.1
3	157	7.7	312	8.3	207	8.8	676	8.3
4	124	6.1	301	8.0	162	6.9	587	7.2
5	195	9.6	395	10.4	250	10.6	840	10.3
6	99	4.9	202	5.3	122	5.2	423	5.2

MZ = Monozygotic, DZ = Dizygotic; PS_SSI_ and PS_SSP_ = Polygenic scores for interest in and probability of same-sex sexual activity, respectively; SSI = Interest in same-sex sexual activity; SSP = Probability of same-sex sexual activity

For MZ twins, DZ twins, siblings and total sample; n for polygenic scores = 939, 1373, 738 and 3050, respectively. Descriptive statistics for SSI and SSP are both reported as continuous^a^ and categorical^b^ variables

#### Phenotypic Correlations

Genetic propensity for same-sex attraction (PS_SSA_) was significantly associated with the same-sex attraction factor (SSA; *r* = 0.06, 95% CI: 0.01–0.10; Tables 2 and 3). The PS_SSA_ factor was significantly correlated with psychological distress (PD: *r* = 0.05; 95% CI: 0.01–0.09) which suggested pleiotropy of PS_SSA_, but PS_SSA_ was not significantly associated with RSB. Same-sex attraction was significantly associated with PD (*r* = 0.25; 95% CIs: 0.22–0.28) and RSB (*r* = 0.28; 95% CIs: 0.26–0.31) which indicated that higher same-sex sexual attraction was associated with higher PD and RSB.

**Table 2:** Factor correlation coefficients and 95% confidence intervals from the phenotypic constrained correlated factors models

Variables	PS_SSA_ (1)	SSA (2)	PD (3a)	RSB (3b)
*Within person*				
1.*^†^	1.00			
2.*^†^	0.06 (0.01, 0.10)	1.00		
3a.*	0.05 (0.01, 0.09)	0.25 (0.22, 0.28)	1.00	
3b.^†^	0.03 (−0.01, 0.06)	0.28 (0.26, 0.31)	–	1.00
Between twins				
*MZ twins*				
1.*^†^	1.00			
2.*^†^	0.06 (0.01, 0.10)	0.71 (0.65, 0.78)		
3a.*	0.05 (0.01, 0.10)	0.21 (0.16, 0.26)	0.52 (0.45, 0.58)	
3b.^†^	0.02 (−0.01, 0.06)	0.21 (0.17, 0.26)	–	0.53 (0.48, 0.58)
*DZ twins*				
1.*^†^	0.50			
2.*^†^	0.03 (−0.02, 0.08)	0.25 (0.20, 0.30)		
3a.*	0.03 (−0.02, 0.08)	0.07 (0.03, 0.11)	0.19 (0.14, 0.24)	
3b.^†^	−0.03 (−0.08, 0.01)	0.09 (0.06, 0.12)	–	0.18 (0.15, 0.22)

PS_SSA_, SSA, PD and RSB = Genetic propensity for same-sex attraction and latent factors for same-sex attraction, psychological distress and risky sexual behavior, respectively; MZ = Monozygotic, DZ = Dizygotic. PS_SSA_ MZ and DZ correlations fixed at 1 and 0.5, respectively

*Phenotypic correlated factors model for PS_SSA_, SSA and PD

^†^Phenotypic correlated factors model for PS_SSA_, SSA and RSB

**Table 3:** Standardized factor loadings and residual path coefficients (with 95% confidence intervals) from the measurement model of the phenotypic constrained correlated factors models

Variables	Factor loadings	Residual path coefficients
PS_SSI_*^†^	0.81 (0.80, 0.82)	0.58 (0.57, 0.60)
PS_SSP_*^†^	1.00 (1.00, 1.00)	0.06 (0.06, 0.07)
SSI*^†^	0.75 (0.73, 0.76)	0.67 (0.65, 0.68)
SSP*^†^	0.73 (0.72, 0.74)	0.68 (0.67, 0.69)
Depressive symptoms*	0.86 (0.85, 0.86)	0.51 (0.50, 0.53)
Anxiety symptoms*	0.76 (0.75, 0.77)	0.65 (0.64, 0.67)
Risky sexual behavior^†a^	1.00	0.00

PS_SSI_ and PS_SSP_ = Polygenic scores for interest in same-sex sexual activity and probability of same-sex sexual activity, respectively; SSI = Interest in same-sex sexual activity; SSP = Probability of same-sex sexual activity

*Phenotypic factor correlation model for PS_SSA_, SSA and PD

^†^Phenotypic factor correlation model for PS_SSA_, SSA and RSB

^a^Factor loading and residual variance for risky sexual behavior were fixed to 1 and 0, respectively, for identification

The cross-twin within-trait correlations of SSA with PD and RSB in monozygotic twins were at least twice those in dizygotic twins indicating only genetic and individual-specific environmental influences on the latent factors. These correlations were consistent with those between the observed variables (Table S1).

#### ACE Influences on Variables and Factors

Consistent with the cross-twin correlations, the variance component influences on the latent factors (Tables 4 and 5)

**Table 4:** Standardized genetic and environmental influences on the variances and covariances of the latent factors (excluding polygenic scores) and 95% confidence intervals from the biometric genetic factor models

	Same-sex attraction (1)	Psychological Distress (2)	Risky sexual behavior (3)
*a*_*f*_^2^			
1.*^†^	0.57 (0.50, 0.64)		
2.*	0.79 (0.61, 0.97)	0.43 (0.36, 0.49)	
3.^†^	0.75 (0.61, 0.88)	–	0.47 (0.42, 0.52)
*c*_*f*_^2^			
1.*^†^	0.00 (0.00, 0.03)		
2.*	0.00 (−0.08, 0.07)	0.00 (0.00, 0.04)	
3.^†^	0.00 (−0.04, 0.05)	–	0.00 (0.00, 0.02)
*e*_*f*_^2^			
1.*^†^	0.43 (0.36, 0.49)		
2.*	0.21 (0.05, 0.39)	0.57 (0.51, 0.63)	
3.^†^	0.25 (0.12, 0.39)	–	0.53 (0.48, 0.58)

*MZ* monozygotic, *DZ* dizygotic; *a*_*f*_^2^, *c*_*f*_^2^ and *e*_*f*_^2^ = Standardized additive genetic and shared and individual-specific environmental influences on the variances (diagonal elements) and covariances between the latent factors

*Biometric genetic model for same-sex attraction and psychological distress

^†^Biometric genetic model for same-sex attraction and risky sexual behavior

**Table 5:** Standardized factor loadings and variable-specific genetic and environmental path coefficients of the indicator variables (excluding the polygenic scores) from the biometric genetic factor models

Variables	Factor loadings	*a*_*s*_	*c*_*s*_	*e*_*s*_
SSI*^†^	0.75 (0.74, 0.76)	0.29 (0.21, 0.34)	0.00 (0.00, 0.17)	0.59 (0.57, 0.62)
SSP*^†^	0.73 (0.72, 0.74)	0.30 (0.21, 0.34)	0.00 (0.00, 0.17)	0.61 (0.59, 0.63)
Depressive symptoms*	0.86 (0.85, 0.86)	0.21 (0.15, 0.24)	0.00 (0.00, 0.11)	0.47 (0.45, 0.49)
Anxiety symptoms*	0.76 (0.75, 0.77)	0.27 (0.19, 0.30)	0.00 (0.00, 0.14)	0.60 (0.58, 0.62)
Risky sexual behavior^†a^	1.00	0.00	0.00	0.00

*MZ* monozygotic, *DZ* dizygotic, *SSI and SSP* interest in and probability of same-sex sexual activity, *a*_*s*_, *c*_*s*_ *and* *e*_*s*_ standardized variable-specific (residual) genetic, shared and individual-specific environmental influences, respectively

^*^Biometric genetic model for same-sex attraction and psychological distress

^†^Biometric genetic model for same-sex attraction and risky sexual behavior

^a^Factor loading and residual variance for risky sexual behavior were fixed to 1 and 0, respectively, for identification

were best resolved into genetic and individual-specific environmental influences. Specifically, the heritability estimates were 57%, 43% and 47% for the same-sex attraction, psychological distress and risky sexual behavior latent factors, respectively (95% CIs: 0.50–0.64, 0.36–0.49 and 0.42–0.52, respectively), while the corresponding standardized individual-specific environmental (E) influences were 43%, 57% and 53% (95% CIs: 0.36–0.49, 0.51–0.63 and 0.48–0.58, respectively). Shared environmental influences on each latent factor were zero (*χ*^2^[8] = 0, *p* = 1) and these components were dropped from subsequent genetic models. These estimates were consistent with those of the observed variables (Table S2) for which heritability estimates ranged between 31% (for depressive symptoms, 95% CI: 0.21–0.37) and 47% (risky sexual behavior, 95% CI: 0.42–0.52) while standardized E influences ranged between 53% (risky sexual behavior, 95% CI: 0.48–0.58) and 67% (depressive symptoms, 95% CI: 0.62–0.73).

#### Mendelian Randomization-Direction of Causation (MRDoC) Models

**Fig. 3:**
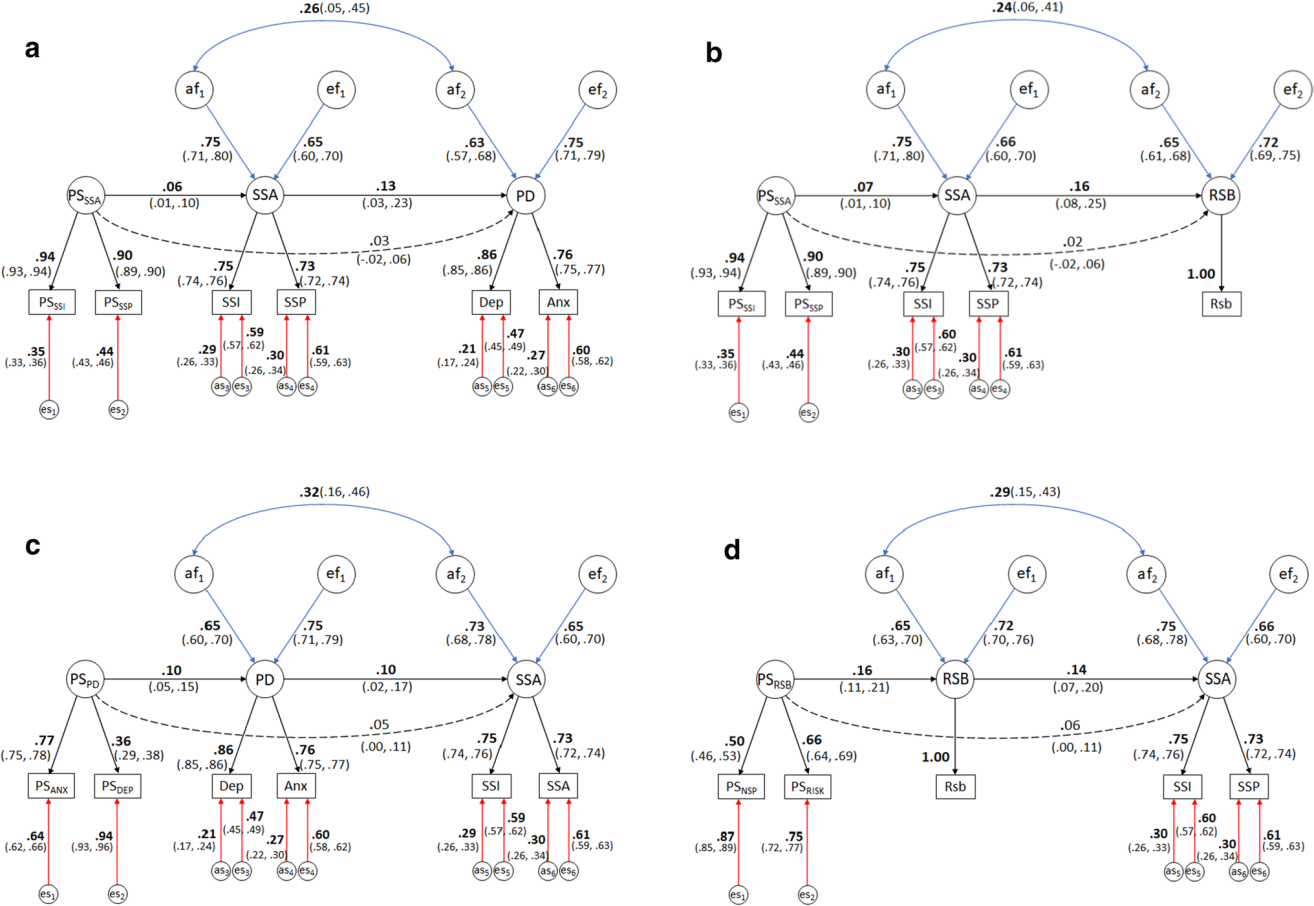
Mendelian Randomization-Direction of Causation models for causal effects of **a** Same-sex attraction (SSA) on psychological distress (PD, Model 1), **b** SSA on risky sexual behavior (RSB, Model 2), **c** PD on SSA (Model 3) and **d** RSB on SSA (Model 4). PS_SSA_ (Genetic propensity for same-sex attraction) included as an instrument for SSA (same-sex attraction latent factor in Models 1 and 2), PS_PD_ (Genetic risk for psychological distress) included as an instrument for PD (psychological distress latent factor in Model 3) and PS_RSB_ (Genetic liability for risky sexual behavior) included as an instrument for RSB (risky sexual behavior latent factor in Model 4). *af*_1_, *af*_2_, *ef*_1_ and *ef*_2_ denote additive genetic and individual-specific environmental influences on the variances predictor and outcome variables, respectively. The correlation between ef_1_ and ef_2_ was omitted to identify the model. *as*_3_–*as*_6_ and *es*_3_–*es*_6_ denote standardized variable-specific additive genetic and individual-specific environmental influences. es_1_ and es_2_ denote the standardized residual variances of PS_SSI_, PS_SSP_, PS_ANX_, PS_DEP_, PS_NSP_ and PS_RISK_ (Polygenic scores for interest in and probability of same-sex sexual activity, anxiety and depressive symptoms, number of sexual partners and risky behaviors, respectively). SSI = Interest in same-sex sexual activity; SSP = Probability of same-sex sexual activity; Dep = Depressive symptoms; Anx = Anxiety symptoms.; Rsb = Risky sexual behavior variable. Broken lines indicate nonsignificant effects (indicated by the 95% CIs straddling zero)

The first MRDoC model (Model 1, with psychological distress as the outcome) indicated a significant causal influence of same-sex attraction on psychological distress which was higher among participants who reported higher same-sex attraction (standardized coefficient: 0.13, 95% CI: 0.03–0.23, *p* = 0.01; Fig. 3). This accounted for 52% of the observed (phenotypic) correlation (*r* = 0.25; Table 2) while the remaining 48% was accounted for by residual genetic correlation. This may also be derived from Fig. 3 by multiplying the coefficients of the paths connecting sexual orientation and psychological distress through their genetic influences (*af*_1_ and *af*_2_, respectively), i.e., 0.75*0.26*0.63 = 0.12 (95% CI: 0.02–0.22) which corresponds to 48% of the observed correlation.

Similarly, same-sex attraction had a significant causal effect on risky sexual behavior (standardized coefficient: 0.16, 95% CI: 0.08–0.25, *p* = 0.0002; Fig. 3) whereby risky sexual behavior was higher among participants who reported greater same-sex attraction. This causal effect accounted for 57% of the phenotypic correlation (*r* = 0.28; Table 2) with residual genetic correlation (0.75*0.24*0.65 = 0.12, 95% CI: 0.03–0.20) explaining the remaining 43%. Taken together, these findings indicate a causal influence of same-sex attraction on psychological distress and risky sexual behavior after controlling for correlated genetic influences.

#### Reverse Causation

The additional MRDoC models (Models 3 and 4; Fig. 3c, d) indicated significant causal influences flowing from psychological distress and risky sexual behavior, respectively, to same-sex attraction (standardized coefficients: 0.10, 0.14; 95% CIs: 0.02–0.17 and 0.07–0.20; *p* = 0.01 and 0.0001, respectively), accounting for 40% and 50% of the respective phenotypic correlations.

### Secondary Analyses

#### Phenotypic Mendelian Randomization Models

The two phenotypic Mendelian Randomization models indicated significant causal influences of same-sex attraction on psychological distress and risky sexual behavior (standardized coefficients = 0.29, 95% CIs: 0.25–0.32; Figures S1 and S2). Although consistent with the findings from the biometric MRDoC models, the causal path coefficients from the phenotypic models were relatively larger. This suggests the possibility of bias from unmeasured confounding (Palmer et al., 2008) and demonstrates the robustness of MRDoC models to multiple sources of bias including pleiotropy and confounding, especially if this is genetic (Minică et al., 2020).

## Discussion

Consistent with existing research, the present study demonstrated that same-sex attraction was associated with greater psychological distress (King et al., 2008; Plöderl & Tremblay, 2015; Semlyen et al., 2016) and risky sexual behavior (Poteat et al., 2019); and that individual differences in same-sex attraction, psychological distress, risky sexual behavior and their relationships could be resolved into genetic and individual-specific components (Burri et al., 2015; Mustanski et al., 2007; Zietsch et al., 2010, 2012). By combining genomic data with traditional biometric twin modeling in the innovative MRDoC model (Minică et al., 2018), the present study for the first time provides empirical evidence for causal influences flowing from same-sex attraction to psychological distress and risky sexual behavior. In addition to this, there were reverse causal paths (i.e., causal influences flowing from psychological distress and risky sexual behavior to same-sex sexual attraction as demonstrated in secondary analyses).

The higher psychological distress and increased risky sexual behavior among same-sex attracted persons are consistent with adverse consequences of sexual minority stress (Meyer, 2013). The genetic influences on these relationships have previously been explained by horizontal pleiotropy such as via common genetic influences on the hypothalamus–pituitary–gonadal and hypothalamus–pituitary–adrenal systems, which, respectively, influence same-sex attraction and depression/anxiety, and may explain their etiological overlap (Zietsch, 2011). However, the coefficients of the pleiotropic paths in all the MRDoC models specified in the present study were of small magnitudes (ranging between 0.02 and 0.06) and not statistically significant (95% confidence intervals ranged between −0.02 and 0.11). This finding suggests that horizontal pleiotropy may not be a sufficient explanation for previously observed genetic correlations between same-sex attraction and adverse health outcomes.

Rather, consistent with prior evidence of vertical pleiotropy (i.e., genetic effects being transmitted through causal paths; Oginni et al., 2020, 2022), the present study demonstrates a phenotypic causal path from same-sex attraction to psychological distress and risky sexual behavior. To our knowledge, this is the first study to provide empirical evidence for this causal relationship which is consistent with the minority stress framework and its extensions. For example, same-sex attraction may engender chronic minority stress which may directly impair cognitive and behavioral self-regulatory processes, resulting in affective and behavioral dysregulation which characterize depressive and anxiety disorders, and risky sexual behaviors, respectively (Hatzenbuehler, 2009). In addition, early rejection and discriminatory experiences, and dispositional characteristics such as neuroticism (Bailey, 2020, 2021) may increase the expectation of such experiences and intensify consequent negative emotional reactions (Feinstein, 2020). More indirectly, same-sex attracted individuals may cope with minority stress using maladaptive strategies which are associated with adverse mental health outcomes (Kaysen et al., 2014; Ngamake et al., 2016) and risky sexual behaviors (Pollard et al., 2018). The causal association between same-sex attraction and risky sexual behavior may also be further mediated by mental health problems including substance use (Oginni et al., 2020, 2022). These may, respectively, reduce self-efficacy in negotiating safe sex practices (Miltz et al., 2017) and increase sexual risk-taking through diminished inhibitions and impaired judgment (Coleman & Cater, 2005; Palamar et al., 2018).

We further demonstrated reverse causal effects being transmitted from psychological distress and risky sexual behavior toward same-sex attraction. A possible explanation for the reverse causal path from psychological distress to same-sex attraction is rejection sensitivity heightening the expectation and perception of rejection in neutral or ambiguous situations (Romero‐Canyas et al., 2010) and/or increasing interpersonal conflicts (Downey & Feldman, 1996; Liu et al., 2014), which may be associated with rejection. Risky sexual behaviors may result in adverse outcomes such as sexually transmitted infection including HIV (Bränström & Pachankis, 2018) which can exacerbate minority stress (Wohl et al., 2013). Thus, we propose individual-specific processes to explain the reverse causal relationships between same-sex attraction, and psychological distress and risky sexual behavior observed in the present study; however, we emphasize that these explanations are speculative and need to be empirically tested.

The findings from the present study thus raise the possibility of a feedback process whereby minority stress experiences associated with same-sex attraction increase the likelihood of adverse mental health outcomes and risky sexual behaviors, which in turn activate processes that maintain or amplify minority stress processes. These latter processes may partly explain the persistent health disparities in same-sex attracted individuals despite increased visibility and tolerance, and legal and social policies to decrease discrimination and promote equality (Meyer et al., 2021). In demonstrating that these relationships are phenotypic (i.e., independent of correlated genetic or environmental etiological influences), the present study indicates that these causal relationships can be completely abolished; however, both causal processes need to be targeted and more research is required to determine the specific mechanisms of the reverse causal relationships. While existing social and legal policies may be intensified to eliminate causal influences flowing from same-sex attraction, reverse causal mechanisms such as rejection sensitivity (e.g., Joss et al., 2020) can be targeted in the assessment and psychotherapeutic agenda for same-sex attracted persons receiving support for psychological distress.

### Conclusion

Our findings provide the first empirical evidence of causal influences flowing from same-sex attraction toward adverse health outcomes including psychological distress and risky sexual behavior. These are consistent with existing evidence and theoretical frameworks implicating minority stress-related processes and provide justification for ongoing efforts aimed at reducing psychosocial disadvantage experienced by sexual minority individuals.

Furthermore, we demonstrated reverse causal influences flowing from psychological distress and risky sexual behavior to same-sex attraction and suggest the possibility of these effects reflecting traits such as rejection sensitivity. Although these causal paths suggest a feedback loop whereby minority stress results in mental health disparities which in turn reinforce minority stress processes, we recognize the possibility that the assessment of same-sex attraction in the present study may be biased by participants’ interest in sex. Our findings highlight the need for further research to clarify the mechanisms of minority stress and mental health disparities among same-sex attracted individuals.

### Strengths and Limitations

The study sample comprised a large nationally representative cohort of twins and their siblings which may increase the generalizability of our findings. Controlling for pleiotropy in the MRDoC model and specifying correlated residual genetic influences also protected against possible associations between instruments and unmeasured confounders. These provided greater rigor in investigating causality compared to standard MR methods.

In interpreting our findings, however, the following limitations need to be considered. Despite the large size of the study sample, only about a third of the participants were genotyped which may worsen the weak instrument bias associated with genetic instruments (Gala & Tomlinson, 2020). As recommended (Gala & Tomlinson, 2020), we compensated for this by incorporating non-twin siblings into the analytic models which increased the number of genotyped participants.

The assessment of same-sex attraction via interest in same-sex sexual activity in the present study may overlap with constructs associated with psychological distress and risky sexual behavior. For example, sensation seeking may be associated with higher same-sex genital responses (Rieger et al., 2013) and more risky sex practices (Roberti, 2004) while recall bias may be implicated in the reverse causal path associated with psychological distress (Heath et al., 1993). The impact of the definition of same-sex attraction is important considering that the assessment of risky sexual behavior in the present study was based on a measure designed to assess sociosexuality—the tendency toward uncommitted sex which is associated with sexual sensation seeking/curiosity (Penke & Asendorpf, 2008), in turn associated with sexual arousal to both sexes (Rieger et al., 2013). Although these other constructs were not included in the present study, uncommitted sexual relationships and multiple sexual partners are recognized HIV sexual risk behaviors (Poteat et al., 2019; Slaymaker, 2004) while same-sex attraction is a reliable indicator of same-sex sexuality (Geary et al., 2018). However, the causal relationships demonstrated in the present study may be further clarified in future studies by combining more specific measures of same-sex sexuality such as sexual identity, sexual fantasies, sexual behavior and sexual attraction (Geary et al., 2018; Smolenski et al., 2010). Similarly, the number of sexual partners can be combined with other sexual risk indicators such as inconsistent condom use and having sex under the influence of psychoactive substances (Cabecinha et al., 2017) in the assessment of risky sexual behavior.

To identify the MRDoC models, we fixed the covariance of residual individual-specific environmental influences on the exposure and outcome variables (r_e_) as is common practice in analyses of twin data (Kohler et al., 2011; Minică et al., 2018). It is also possible that our estimates of the causal path coefficients were biased by this parameter. As recommended (Minică et al., 2018), we varied the fixed value of r_e_ and observed changes in the causal path coefficients. However, the model fit indices of these models were identical and did not allow for any meaningful inferences. A previous study showed that estimates of causal effects derived from an MRDoC model in which r_e_ was dropped was more conservative and less biased compared with those from a standard MR model (Minică et al., 2020). This suggests that the impact of dropping r_e_ from the MRDoC model is likely to be minimal. Related to this, while we interpreted residual genetic correlations as indicating reverse vertical pleiotropy, this was not specified in the index model, but detected in alternative models; it is possible that there is some residual genetic correlation in addition to the forward and reverse phenotypic causal paths. A better alternative model to detect this residual correlation would be a bidirectional MRDoC model which specifies both (forward and reverse) causal paths and incorporates both instruments in a single model. However, specifying this model in exploratory analyses yielded an unstable model with unreliable causal path estimates. We attributed this to the unbalanced pleiotropy between the instruments (i.e., pleiotropy between same-sex attraction and PS_PD_ and PS_RSB_ was greater compared to that between PS_SSA_ and psychological distress and risky sexual behavior). An ideal bidirectional MRDoC model would potentially utilize non-pleiotropic instruments (Heath et al., 1993), but this still requires further methodological work. Though the MRDoC tests for causality using cross-sectional data, it is possible there is still some unmeasured bias. Thus, it is important to replicate our findings using alternative methods for causal inference (Minică et al., 2018) such as longitudinal designs with appropriate statistical modeling.

MR and its derivatives assume linear relationships between the instrument, exposure and outcome (Gala & Tomlinson, 2020); however, the higher rates of psychological distress and risky sexual behavior among bisexual relative to exclusively same-sex attracted and heterosexual individuals (Poteat et al., 2019; Ross et al., 2018; Wicki et al., 2021) raise the possibility of nonlinear causal relationships stronger for bisexual relative to more exclusively same-sex attracted and heterosexual persons. Future studies may investigate this by using nonlinear Mendelian Randomization approaches (e.g., Sun et al., 2019) which may include stratifying by sexual orientation and analyzing subgroups. Considering previously reported sex differences in the mental health disparities among sexual minorities (King et al., 2008; Plöderl & Tremblay, 2015) and the genetic influences on these (Ganna et al., 2019); investigating sex differences in these causal relationships can also help in identifying vulnerable groups who can be differentially targeted for interventions. Such sex differences were not investigated in the present study because the already wide confidence intervals indicated a low power to test sex differences when male are compared against female participants.

Finally, the analytic models used in the present study do not indicate specific psychopathogenic processes. While we have drawn extensively on the minority stress model (Meyer, 2013) and its derivatives (Feinstein, 2020; Hatzenbuehler, 2009); it is possible that there are other mechanisms for the health disparities observed in same-sex attracted relative to heterosexual individuals which have yet to be identified. As such, our demonstration of non-genetic causal pathways for these disparities provides a justification for continued research to elucidate these mechanisms while indicating the possibility that identified mechanisms can be targeted for individual- and social-level interventions.

## Supplementary Information

Below is the link to the electronic supplementary material.

Supplementary file1 (DOCX 69 kb)

## Data Availability

Not publicly available.

## Code Availability

Available on request.
