# Supplementary material for: Causal Influences of Same-Sex Attraction on Psychological Distress and Risky Sexual Behaviors: Evidence for Bidirectional Effects
Source: Arch Sex Behav. 2022 Nov 4;52(3):1213–28. doi: 10.1007/s10508-022-02455-9 (PMC10102149; doi:10.1007/s10508-022-02455-9)
Supplement: Supplementary file 1 — Supplementary file1 (DOCX 69 kb) [file 10508_2022_2455_MOESM1_ESM.docx]

Supplementary material for

**Causal Influences of Same-Sex Attraction on Psychological Distress and Risky Sexual Behaviors: Evidence for Bidirectional Effects**

**Method**

***Quality control for single nucleotide polymorphisms (SNPs) and sample***

Quality control (QC) was carried out using RICOPILI (Rapid Imputation for COnsortias PIpeLIne) – an open -sourced Perl-based pipeline (Lam et al., 2020) and post-QC imputation was carried out using the Sanger imputation service to the Haplotype Reference Consortium reference panel (McCarthy et al., 2016). Eagle2 and IMPUTE version 2 were used for prephasing and imputing respectively (Howie et al, 2009). Specific sample QC parameters included sex inconsistencies (17 participants excluded), genotyping efficiency (call rate < 0.05, 110 participants excluded), autosomal heterozygosity deviation (threshold set at < 0.2 – 14 participants excluded), ancestry was restricted to participants of European descent and 2 outliers were excluded based on 20 principal components (Lam et al., 2020). SNP QC parameters included SNP missingness < 0.05 before sample removal (29196 SNPs excluded – 4%), SNP missingness < 0.02 after sample removal (24106 SNPs – 3.3%) and SNP Hardy-Weinberg equilibrium (p > 10^-6^, 18781 SNPs – 2.8%).

**Results**

**Table S0:**

*PRSice-2 polygenic score prediction results*

| Polygenic score | Target phenotype | Threshold | R^2^ | No of SNPs | Empirical *p* |
| --- | --- | --- | --- | --- | --- |
| ^a^PS_SSI_ | SSI | 6.00x10^-4^ | 4.60x10^-3^ | 136 | 3.70x10^-3^ |
| ^a^PS_SSP_ | SSP | 9.50x10^-4^ | 3.43x10^-3^ | 214 | 1.44x10^-2^ |
| ^b^PS_DEP_ | Dep | 1.11x10^-2^ | 6.09x10^-3^ | 4547 | 6.00x10^-4^ |
| ^b^PS_ANX_ | Anx | 2.81x10^-2^ | 2.87x10^-3^ | 5730 | 3.95x10^-2^ |
| ^b^PS_NSP_ | RSB | 5.50x10^-4^ | 1.16x10^-1^ | 1085 | 9.99x10^-5^ |
| ^b^PS_RISK_ | RSB | 1.16x10^-1^ | 8.60x10^-3^ | 21879 | 9.99x10^-5^ |

*Note.* Threshold = *p*-value threshold for SNP inclusion that maximized variance explained in the target phenotype; R^2^ = variance in target phenotype explained by the polygenic score; No. of SNPs = number of SNPs included in the polygenic score; Empirical *p* is the *p*-value obtained from 100,000 permutations which corrects for data overfitting and multiple testing to obtain the optimal threshold; PS_SSI_, PS_SSP_, PS_DEP_, PS_ANX_, PS_NSP_ and PS_RISK_ = Polygenic scores for interest in same-sex sexual activity, probability of same-sex sexual activity, depressive symptoms, anxiety symptoms, number of sexual partners and risky behaviors respectively; SSI = Interest in same-sex sexual activity; SSP = Probability of same-sex sexual activity; Dep and Anx = Depressive and anxiety symptoms respectively; RSB = Risky sexual behavior.

^a^Main analyses.

^b^Secondary analyses.

**Table S1**

*Within-person and between-twin correlations of the study variables and 95% confidence intervals from the phenotypic constrained correlation model*

| Variables | PS_SSI_  (1) | PS_SSP_  (2) | SSI  (3) | SSP  (4) | Depressive symptoms (5) | Anxiety symptoms  (6) | RSB  (7) |
| --- | --- | --- | --- | --- | --- | --- | --- |
| Within person |  |  |  |  |  |  |  |
| 1. | 1.00 |  |  |  |  |  |  |
| 2. | 0.83  (0.82, 0.83) | 1.00 |  |  |  |  |  |
| 3. | 0.04  (0.04, 0.04) | 0.05  (0.05, 0.05) | 1.00 |  |  |  |  |
| 4. | 0.05  (0.05, 0.05) | 0.04  (0.04, 0.04) | 0.55  (0.55, 0.55) | 1.00 |  |  |  |
| 5. | 0.02  (0.02, 0.02) | 0.04  (0.04, 0.04) | 0.15  (0.15, 0.15) | 0.16  (0.16, 0.16) | 1.00 |  |  |
| 6. | 0.04  (0.04, 0.04) | 0.05  (0.05, 0.05) | 0.15  (0.15, 0.15) | 0.14  (0.14, 0.14) | 0.65  (0.65, 0.65) | 1.00 |  |
| 7. | 0.03  (0.03, 0.03) | 0.03  (0.03, 0.03) | 0.18  (0.18, 0.18) | 0.24  (0.24, 0.24) | 0.13  (0.13, 0.13) | 0.09  (0.09, 0.09) | 1.00 |
| Between twin |  |  |  |  |  |  |  |
| MZ twins |  |  |  |  |  |  |  |
| 1. | 1.00  (1.00, 1.00) |  |  |  |  |  |  |
| 2. | 0.83  (0.82, 0.83) | 1.00  (1.00, 1.00) |  |  |  |  |  |
| 3. | 0.04  (0.04, 0.04) | 0.05  (0.05, 0.05) | 0.41  (0.41, 0.41) |  |  |  |  |
| 4. | 0.04  (0.04, 0.04) | 0.04  (0.04, 0.04) | 0.35  (0.35, 0.35) | 0.45  (0.45, 0.45) |  |  |  |
| 5. | 0.02  (0.02, 0.02) | 0.04  (0.04, 0.04) | 0.13  (0.13, 0.13) | 0.14  (0.14, 0.14) | 0.37  (0.37, 0.37) |  |  |
| 6. | 0.04  (0.04, 0.04) | 0.05  (0.05, 0.05) | 0.12  (0.12, 0.12) | 0.11  (0.11, 0.11) | 0.33  (0.33, 0.33) | 0.41  (0.41, 0.41) |  |
| 7. | 0.03  (0.03, 0.03) | 0.02  (0.02, 0.02) | 0.15  (0.15, 0.15) | 0.17  (0.17, 0.17) | 0.09  (0.09, 0.09) | 0.08  (0.08, 0.08) | 0.53  (0.53, 0.53) |
| DZ twins |  |  |  |  |  |  |  |
| 1. | 0.55  (0.55, 0.55) |  |  |  |  |  |  |
| 2. | 0.44  (0.44, 0.44) | 0.52  (0.52, 0.52) |  |  |  |  |  |
| 3. | 0.03  (0.03, 0.03) | 0.04  (0.04, 0.04) | 0.16  (0.16, 0.16) |  |  |  |  |
| 4. | 0.03  (0.03, 0.03) | 0.02  (0.02, 0.02) | 0.11  (0.11, 0.11) | 0.15  (0.15, 0.15) |  |  |  |
| 5. | 0.02  (0.02, 0.02) | 0.03  (0.03, 0.03) | 0.06  (0.06, 0.06) | 0.01  (0.01, 0.01) | 0.13  (0.13, 0.13) |  |  |
| 6. | 0.02  (0.02, 0.02) | 0.03  (0.03, 0.03) | 0.08  (0.08, 0.08) | 0.03  (0.03, 0.03) | 0.11  (0.11, 0.11) | 0.14  (0.14, 0.14) |  |
| 7. | -0.04  (-0.04, -0.04) | -0.03  (-0.03, -0.03) | 0.06  (0.06, 0.06) | 0.07  (0.07, 0.07) | 0.02  (0.02, 0.02) | 0.04  (0.04, 0.04) | 0.18  (0.18, 0.18) |

*Note.* MZ = Monozygotic, DZ = Dizygotic; PS_SSI_ and PS_SSP_ = Polygenic scores for interest in same-sex sexual activity and probability of same-sex sexual activity respectively; SSI = Interest in same-sex sexual activity; SSP = Probability of same-sex sexual activity; RSB = Risky sexual behavior.

**Table S2**

*Standardized genetic and environmental influences on the variances and covariances between the study variables (excluding the polygenic scores) and 95% confidence intervals from the multivariate biometric genetic models*

| Variables | SSI  (1) | SSP  (2) | Depressive symptoms  (3) | Anxiety symptoms  (4) | RSB  (5) |
| --- | --- | --- | --- | --- | --- |
| h^2^ |  |  |  |  |  |
| 1.*† | 0.37  (0.27, 0.43) |  |  |  |  |
| 2.*† | 0.60  (0.50, 0.68) | 0.39  (0.32, 0.45) |  |  |  |
| 3.* | 0.75  (0.38, 1.15) | 0.80  (0.51, 1.16) | 0.31  (0.21, 0.37) |  |  |
| 4.* | 0.79  (0.40, 1.08) | 0.79  (0.47, 1.12) | 0.43  (0.33, 0.51) | 0.35  (0.28, 0.41) |  |
| 5.† | 0.82  (0.58, 1.06) | 0.69  (0.54, 0.83) | - | - | 0.47  (0.42, 0.52) |
|  |  |  |  |  |  |
| c^2^ |  |  |  |  |  |
| 1.*† | 0.02  (0.00, 0.08) |  |  |  |  |
| 2.*† | -0.02  (-0.06, 0.04) | 0.01  (0.00, 0.06) |  |  |  |
| 3.* | 0.09  (-0.14, 0.31) | -0.10  (-0.31, 0.04) | 0.02  (0.00, 0.08) |  |  |
| 4.* | 0.08  (-0.06, 0.32) | -0.06  (-0.23, 0.12) | 0.02  (-0.02, 0.08) | 0.01  (0.00, 0.05) |  |
| 5.† | -0.01  (-0.13, 0.12) | 0.00  (-0.04, 0.07) | - | - | 0.00  (0.00, 0.02) |
|  |  |  |  |  |  |
| e^2^ |  |  |  |  |  |
| 1.*† | 0.61  (0.57, 0.67) |  |  |  |  |
| 2.*† | 0.42  (0.35, 0.49) | 0.59  (0.55, 0.64) |  |  |  |
| 3.* | 0.16  (-0.09, 0.39) | 0.30  (0.08, 0.51) | 0.67  (0.62, 0.73) |  |  |
| 4.* | 0.13  (-0.09, 0.36) | 0.27  (0.03, 0.51) | 0.55  (0.49, 0.62) | 0.64  (0.59, 0.69) |  |
| 5.† | 0.18  (0.00, 0.37) | 0.30  (0.17, 0.44) | - | - | 0.53  (0.48, 0.58) |

*Note*. SSI = Interest in same-sex sexual activity; SSP = Probability of same-sex sexual activity; RSB = Risky sexual behavior; h^2^, c^2^ and e^2^ = Standardized additive genetic and shared and individual-specific environmental influences on the variances (diagonal elements) and covariances of the study variables (excluding polygenic scores). *SSI, SSP and depressive and anxiety symptoms included in this biometric genetic model. †SSI, SSP and RSB included in this biometric genetic model.

**Figure S1**

*The phenotypic Mendelian Randomization model 1 (with Psychological distress as the outcome)*


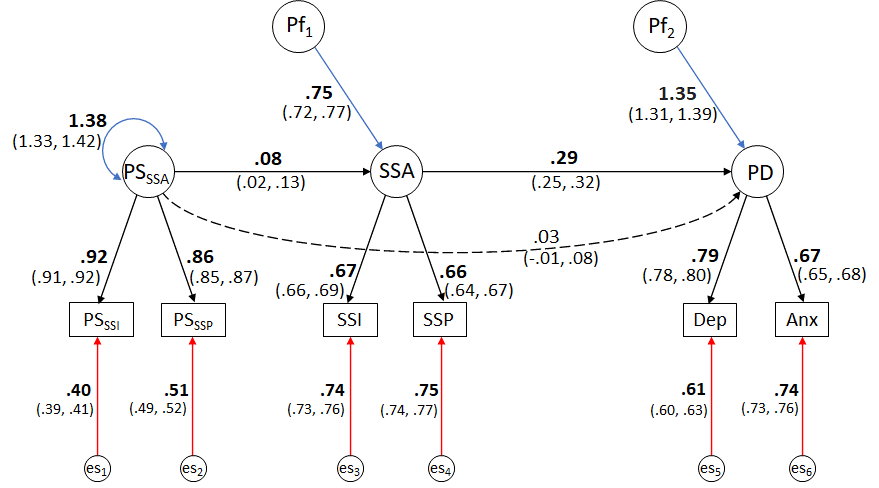


*Note*. Instrumental, pleiotropic and causal paths from PS_SSA_ (Genetic propensity for same-sex attraction) to SSA (Same-sex attraction latent factor), PS_SSA_ to PD (Psychological distress latent factor) and SSA to PD respectively. Pf_1_ and Pf_2_ denote residual variances for SSA and PD respectively. es_1_-es_6_ denote variable-specific residual variances. PS_SSI_ and PS_SSP_ = Polygenic scores for interest in same-sex sexual activity and probability of same-sex sexual activity respectively; SSI = Interest in same-sex sexual activity; SSP = Probability of same-sex sexual activity; Dep = Depressive symptoms; Anx = Anxiety symptoms. Broken lines indicate non-significant effects (indicated by 95% Cis straddling zero).

**Figure S2**

*The phenotypic Mendelian Randomization model 2 (with Risky sexual behavior as the outcome)*


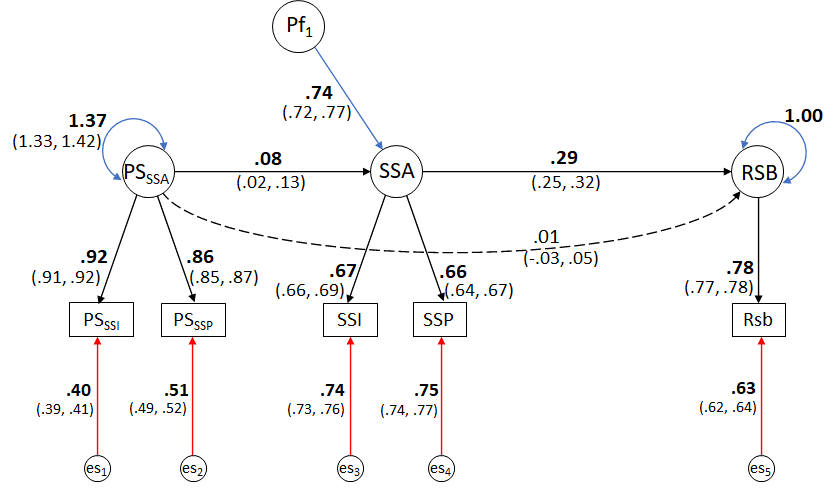


*Note*. Instrumental, pleiotropic and causal paths from PS_SSA_ (Genetic propensity for same-sex attraction) to SSA (Same-sex attraction latent factor), PS_SSA_ to RSB (Risky sexual behavior latent factor) and SSA to RSB respectively. Pf_1_ denotes residual variance for SSA. es_1_-es_5_ denote variable-specific residual variances. PS_SSI_ and PS_SSP_ = Polygenic scores for interest in same-sex sexual activity and probability of same-sex sexual activity respectively; SSI = Interest in same-sex sexual activity; SSP = Probability of same-sex sexual activity; Rsb = Risky sexual behavior variable. Broken lines indicate non-significant effects (indicated by 95% CIs straddling zero).

**References**

Lam, M., Awasthi, S., Watson, H. J., Goldstein, J., Panagiotaropoulou, G., Trubetskoy, V., Karlsson, R., Frei, O., Fan, C.-C., & De Witte, W. (2020). RICOPILI: Rapid imputation for COnsortias PIpeLIne. *Bioinformatics, 36*(3), 930-933. <https://doi.org/10.1093/bioinformatics/btz633>

McCarthy, S., Das, S., Kretzschmar, W., Delaneau, O., Wood, A. R., Teumer, A., Kang, H. M., Fuchsberger, C., Danecek, P., Sharp, K., Luo, Y., Sidore, C., Kwong, A., Timpson, N., Koskinen, S., Vrieze, S., Scott, L. J., Zhang, H., Mahajan, A., . . . Marchini, J. (2016). A reference panel of 64,976 haplotypes for genotype imputation. *Nature Genetics, 48*(10), 1279-1285. https://doi.org/10.1038/ng.3643
